# [*S*-Allyl-4-(4-hy­droxy-2-oxidobenzyl­idene-κ*O*)-1-(2-oxidobenzyl­idene-κ*O*)isothio­semicarbazidato-κ^2^ *N* ^1^,*N* ^4^](ethanol-κ*O*)dioxido­uranium(VI) ethanol monosolvate

**DOI:** 10.1107/S1600536811049579

**Published:** 2011-11-25

**Authors:** Reza Takjoo, Grzegorz Dutkiewicz, Maciej Kubicki

**Affiliations:** a Department of Chemistry, School of Sciences, Ferdowsi University of Mashhad, Mashhad 91775-1436, Iran; b Department of Chemistry, Adam Mickiewicz University, Grunwaldzka 6, 60-780 Poznań, Poland

## Abstract

In the title compound, [U(C_18_H_15_N_3_O_3_S)O_2_(C_2_H_5_OH)]·C_2_H_5_OH, the U^VI^ ion is in a distorted penta­gonal–bipyramidal coordination geometry, with two oxide O atoms in axial sites. Two N and two O atoms of the tetra­dentate ligand and an O atom of an ethanol ligand form the equatorial plane. The dihedral angle between the mean planes of the two benzene rings is 34.8 (3)°. In the crystal, relatively strong O—H⋯O hydrogen bonds connect the complex and ethanol solvent mol­ecules into alternating centrosymmetric *R*_2_^2^(8) and *R*_4_^4^(16) ring motifs, forming chains along [100]. Weak inter­molecular C—H⋯O hydrogen bonds are also present.

## Related literature

For background information on salicyl­aldehyde-*S*-alkyl-thio­semicarbazone compounds, see: Gerbeleu & Revenko (1971 ▶); Revenko *et al.* (1986 ▶); Simonov *et al.* (1985 ▶); Yampol’skaya *et al.* (1982 ▶, 1983 ▶). For a related structure, see: Kawasaki & Kitazawa (2008 ▶). For hydrogen-bond motifs, see: Bernstein *et al.* (1995 ▶).
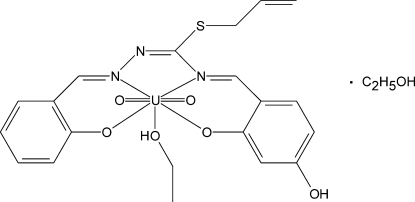


## Experimental

### 

#### Crystal data

[U(C_18_H_15_N_3_O_3_S)O_2_(C_2_H_6_O)]·C_2_H_6_O
*M* *_r_* = 715.56
Monoclinic, 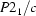

*a* = 12.0152 (4) Å
*b* = 17.6609 (4) Å
*c* = 13.1076 (3) Å
β = 109.195 (3)°
*V* = 2626.79 (12) Å^3^
*Z* = 4
Mo *K*α radiation
μ = 6.30 mm^−1^
*T* = 100 K
0.20 × 0.12 × 0.10 mm

#### Data collection

Agilent Xcalibur Eos diffractometer
Absorption correction: multi-scan (*CrysAlis PRO*; Agilent, 2011 ▶) *T*_min_ = 0.424, *T*_max_ = 1.000
11844 measured reflections
5467 independent reflections
4148 reflections with *I* > 2σ(*I*)
*R*_int_ = 0.031

#### Refinement

*R*[*F* ^2^ > 2σ(*F* ^2^)] = 0.041
*wR*(*F* ^2^) = 0.120
*S* = 1.01
5467 reflections
309 parameters
1 restraint
H-atom parameters constrained
Δρ_max_ = 2.48 e Å^−3^
Δρ_min_ = −1.18 e Å^−3^

### 

Data collection: *CrysAlis PRO* (Agilent, 2011 ▶); cell refinement: *CrysAlis PRO*; data reduction: *CrysAlis PRO*; program(s) used to solve structure: *SIR92* (Altomare *et al.*, 1993 ▶); program(s) used to refine structure: *SHELXL97* (Sheldrick, 2008 ▶); molecular graphics: *SHELXTL* (Sheldrick, 2008 ▶) and *Mercury* (Macrae *et al.*, 2008 ▶); software used to prepare material for publication: *SHELXL97*.

## Supplementary Material

Crystal structure: contains datablock(s) global. DOI: 10.1107/S1600536811049579/lh5378sup1.cif

Additional supplementary materials:  crystallographic information; 3D view; checkCIF report

## Figures and Tables

**Table 1:** Hydrogen-bond geometry (Å, °)

*D*—H⋯*A*	*D*—H	H⋯*A*	*D*⋯*A*	*D*—H⋯*A*
O4—H4⋯O8^i^	0.86	1.75	2.601 (7)	173
O29—H29⋯O1*S*	0.86	1.79	2.646 (9)	171
O1*S*—H1*S*1⋯O26^ii^	0.86	1.87	2.718 (8)	169
C23—H23⋯O2^iii^	0.95	2.49	3.395 (9)	158
C27—H27⋯O1*S*	0.95	2.51	3.189 (10)	128

Symmetry codes: (i) 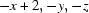; (ii) 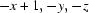; (iii) 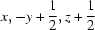.

**Fig. 1.**
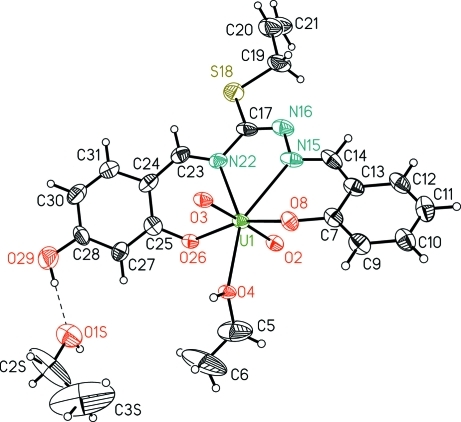
Anisotropic ellipsoid representation of (I) with ellipsoids drawn at 50% probability level. Hydrogen atoms are depicted as spheres with arbitrary radii.

**Fig. 2.**
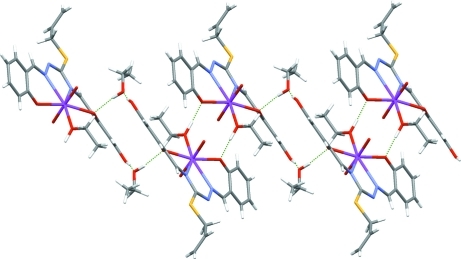
A hydrogen bonded (dotted lines) chain along [100].

## supplementary crystallographic information

### Comment

Some salicylaldehyde-*S*-alkyl-thiosemicarbazones have been synthesized by template condensation of *S*-alkylisothiosemicarbazide with related aldehydes and ketones. These compounds were synthesized and characterized for the first time by Gerbeleu & Revenko (1971) and a few studies of these compounds have been carried out (*e.g.* Yampol'skaya *et al.*, 1982, 1983; Simonov *et al.*, 1985, Revenko *et al.*, 1986). The fundamental investigations of the bonding and structure of uranium complexes provides important information on the field of backend chemistry (Kawasaki & Kitazawa, 2008). We report herein the synthesis and crystal structure of the title compound (I).

The molecular structure of (I) is shown in Fig. 1. The ligand takes part in the coordination to uranium as a double-deprotonated tetradentate N_2_O_2_ ligand (maximum deviation from the least-squares plane by four coordinating atoms is 0.35 Å). The coordination is a quite distorted pentagonal bipyramid with two oxo O atoms in the axial sites, and the pentagonal equatorial plane, which is relatively far from planarity, is completed by an ethanol ligand. The ligand molecule itself is slightly folded as the dihedral angle between the benzene ring planes is 34.8 (3)°; the C═CH_2_ group is almost perpendicular to the mean ring plane (S18—C19—C20═C21 torsion angle = -100.9 (10)°).

In the crystal, relatively strong and directional O—H···O hydrogen bonds join the complex molecules into dimers which are further expanded into a chain. There are two different centrosymmetric dimers, one created by the O4—H4···O8(2 - *x*,-*y*,-*z*) interactions, with the graph set *R*^2^_2_(8) (Bernstein *et al.*, 1995) and the other formed by O29—H29···O1S and O1S—H1S···O26 (1-*x*,-*y*,-*z*), with the graph set *R*^4^_4_(16). The sequence of these dimers creates a hydrogen-bonded chain (Fig. 2).

### Experimental

UO_2_(OAc)_2_.2H_2_O (0.42 g, 1.0 mmol) in 20 cm^3^ ethanol was added to a solution of salicylaldehyde mono-*S*-allylisothiosemicarbazone hydrobromide (0.32 g, 1.0 mmol), 2,4-dihydroxybenzaldehyde (0.14 g, 1.0 mmol) and 0.35 cm^3^ (2.5 mmol) triethylamine. This red solution was heated under reflux for 1 h at 343K. Red crystals appeared after 4 days then were collected by filtration, washed with ethanol, and dried in air.

### Refinement

Hydrogen atoms were generated geometrically and refined as a riding model with their *U*_iso_(H) set at 1.2-1.5 times *U*_eq_ of appropriate carrier atom. A weak distance constraint were applied to the C—C distance in the coordinated ethanol molecule.

### Crystal data

**Table:**

[U(C_18_H_15_N_3_O_3_S)O_2_(C_2_H_6_O)]·C_2_H_6_O	*F*(000) = 1376
*M**_r_* = 715.56	*D*_x_ = 1.809 Mg m^−^^3^
Monoclinic, *P*2_1_/*c*	Mo *K*α radiation, λ = 0.71073 Å
Hall symbol: -P 2ybc	Cell parameters from 4645 reflections
*a* = 12.0152 (4) Å	θ = 2.8–28.2°
*b* = 17.6609 (4) Å	µ = 6.30 mm^−^^1^
*c* = 13.1076 (3) Å	*T* = 100 K
β = 109.195 (3)°	Block, red
*V* = 2626.79 (12) Å^3^	0.2 × 0.12 × 0.1 mm
*Z* = 4	

### Data collection

**Table:**

Agilent Xcalibur Eos diffractometer	5467 independent reflections
Radiation source: Enhance (Mo) X-ray Source	4148 reflections with *I* > 2σ(*I*)
graphite	*R*_int_ = 0.031
Detector resolution: 16.1544 pixels mm^-1^	θ_max_ = 28.3°, θ_min_ = 2.9°
ω–scan	*h* = −15→15
Absorption correction: multi-scan (*CrysAlis PRO*; Agilent, 2011)	*k* = −22→21
*T*_min_ = 0.424, *T*_max_ = 1.000	*l* = −16→16
11844 measured reflections	

### Refinement

**Table:**

Refinement on *F*^2^	Primary atom site location: structure-invariant direct methods
Least-squares matrix: full	Secondary atom site location: difference Fourier map
*R*[*F*^2^ > 2σ(*F*^2^)] = 0.041	Hydrogen site location: inferred from neighbouring sites
*wR*(*F*^2^) = 0.120	H-atom parameters constrained
*S* = 1.01	*w* = 1/[σ^2^(*F*_o_^2^) + (0.070*P*)^2^ + 2.0206*P*] where *P* = (*F*_o_^2^ + 2*F*_c_^2^)/3
5467 reflections	(Δ/σ)_max_ = 0.001
309 parameters	Δρ_max_ = 2.48 e Å^−^^3^
1 restraint	Δρ_min_ = −1.18 e Å^−^^3^

### Special details

**Table:**

Geometry. All s.u.'s (except the s.u. in the dihedral angle between two l.s. planes) are estimated using the full covariance matrix. The cell s.u.'s are taken into account individually in the estimation of s.u.'s in distances, angles and torsion angles; correlations between s.u.'s in cell parameters are only used when they are defined by crystal symmetry. An approximate (isotropic) treatment of cell s.u.'s is used for estimating s.u.'s involving l.s. planes.
Refinement. Refinement of *F*^2^ against ALL reflections. The weighted *R*-factor *wR* and goodness of fit *S* are based on *F*^2^, conventional *R*-factors *R* are based on *F*, with *F* set to zero for negative *F*^2^. The threshold expression of *F*^2^ > σ(*F*^2^) is used only for calculating *R*-factors(gt) *etc*. and is not relevant to the choice of reflections for refinement. *R*-factors based on *F*^2^ are statistically about twice as large as those based on *F*, and *R*- factors based on ALL data will be even larger.

### Fractional atomic coordinates and isotropic or equivalent isotropic displacement parameters (Å^2^)

**Table:**

	*x*	*y*	*z*	*U*_iso_*/*U*_eq_	
U1	0.85612 (2)	0.123346 (13)	−0.019954 (19)	0.02921 (11)	
O2	0.7621 (4)	0.1424 (3)	−0.1531 (4)	0.0344 (11)	
O3	0.9470 (4)	0.1011 (3)	0.1138 (4)	0.0347 (11)	
O4	0.8352 (4)	−0.0092 (2)	−0.0602 (4)	0.0310 (10)	
H4	0.8855	−0.0413	−0.0219	0.037*	
C5	0.7523 (9)	−0.0468 (5)	−0.1482 (8)	0.084 (4)	
H5A	0.7917	−0.0624	−0.2003	0.101*	
H5B	0.6887	−0.0108	−0.1856	0.101*	
C6	0.6985 (12)	−0.1151 (6)	−0.1151 (12)	0.122 (7)	
H6A	0.7606	−0.1518	−0.0801	0.183*	
H6B	0.6417	−0.1383	−0.1790	0.183*	
H6C	0.6581	−0.1000	−0.0644	0.183*	
C7	1.0608 (6)	0.1338 (4)	−0.1445 (6)	0.0354 (17)	
O8	1.0218 (5)	0.1062 (3)	−0.0663 (4)	0.0356 (11)	
C9	1.1165 (6)	0.0868 (4)	−0.1974 (6)	0.0384 (17)	
H9	1.1250	0.0344	−0.1804	0.046*	
C10	1.1594 (7)	0.1156 (4)	−0.2739 (7)	0.0441 (19)	
H10	1.1980	0.0828	−0.3090	0.053*	
C11	1.1473 (7)	0.1928 (5)	−0.3016 (6)	0.048 (2)	
H11	1.1760	0.2120	−0.3560	0.057*	
C12	1.0942 (7)	0.2394 (4)	−0.2495 (6)	0.0431 (19)	
H12	1.0883	0.2918	−0.2666	0.052*	
C13	1.0469 (6)	0.2121 (4)	−0.1696 (6)	0.0379 (17)	
C14	0.9981 (6)	0.2648 (4)	−0.1165 (6)	0.0397 (18)	
H14	1.0081	0.3165	−0.1315	0.048*	
N15	0.9399 (5)	0.2520 (3)	−0.0483 (5)	0.0397 (15)	
N16	0.9061 (6)	0.3185 (4)	−0.0119 (6)	0.0482 (17)	
C17	0.8425 (7)	0.3098 (4)	0.0480 (6)	0.0363 (17)	
S18	0.7961 (2)	0.39101 (11)	0.09995 (18)	0.0496 (5)	
C19	0.8632 (9)	0.4648 (4)	0.0437 (8)	0.059 (2)	
H19A	0.9477	0.4536	0.0582	0.070*	
H19B	0.8241	0.4678	−0.0355	0.070*	
C20	0.8496 (9)	0.5379 (5)	0.0953 (8)	0.057 (2)	
H20	0.9020	0.5486	0.1658	0.068*	
C21	0.7689 (9)	0.5878 (5)	0.0483 (8)	0.065 (3)	
H21A	0.7155	0.5784	−0.0222	0.078*	
H21B	0.7637	0.6336	0.0844	0.078*	
N22	0.8004 (5)	0.2383 (3)	0.0693 (5)	0.0353 (14)	
C23	0.7552 (6)	0.2332 (4)	0.1475 (6)	0.0395 (18)	
H23	0.7568	0.2779	0.1883	0.047*	
C24	0.7047 (7)	0.1687 (5)	0.1774 (6)	0.0411 (18)	
C25	0.6751 (6)	0.1012 (4)	0.1140 (6)	0.0337 (16)	
O26	0.6906 (4)	0.0979 (3)	0.0188 (4)	0.0325 (11)	
C27	0.6239 (7)	0.0409 (4)	0.1483 (6)	0.0379 (17)	
H27	0.6041	−0.0036	0.1056	0.046*	
C28	0.6012 (7)	0.0454 (4)	0.2465 (6)	0.0431 (19)	
O29	0.5576 (5)	−0.0114 (4)	0.2859 (5)	0.0623 (17)	
H29	0.5365	−0.0487	0.2416	0.075*	
C30	0.6283 (8)	0.1125 (5)	0.3084 (7)	0.054 (2)	
H30	0.6127	0.1160	0.3747	0.065*	
C31	0.6763 (7)	0.1708 (5)	0.2730 (6)	0.0443 (19)	
H31	0.6919	0.2160	0.3146	0.053*	
O1S	0.5141 (6)	−0.1238 (3)	0.1448 (6)	0.0641 (18)	
H1S1	0.4504	−0.1215	0.0902	0.077*	
C2S	0.5324 (11)	−0.1992 (7)	0.1650 (15)	0.131 (7)	
H2S1	0.4597	−0.2228	0.1700	0.157*	
H2S2	0.5958	−0.2066	0.2351	0.157*	
C3S	0.5674 (15)	−0.2376 (10)	0.0748 (16)	0.187 (10)	
H3S1	0.6259	−0.2064	0.0570	0.280*	
H3S2	0.4975	−0.2434	0.0106	0.280*	
H3S3	0.6011	−0.2876	0.0994	0.280*	

### Atomic displacement parameters (Å^2^)

**Table:**

	*U*^11^	*U*^22^	*U*^33^	*U*^12^	*U*^13^	*U*^23^
U1	0.03164 (17)	0.02203 (15)	0.03026 (15)	0.00084 (11)	0.00514 (11)	0.00180 (10)
O2	0.037 (3)	0.027 (2)	0.037 (3)	0.002 (2)	0.010 (2)	0.004 (2)
O3	0.037 (3)	0.027 (2)	0.038 (3)	0.001 (2)	0.010 (2)	0.002 (2)
O4	0.030 (3)	0.026 (2)	0.030 (2)	0.002 (2)	0.001 (2)	−0.001 (2)
C5	0.084 (8)	0.055 (6)	0.074 (7)	−0.001 (6)	−0.026 (6)	−0.012 (5)
C6	0.081 (10)	0.057 (7)	0.168 (16)	−0.022 (6)	−0.042 (10)	−0.001 (7)
C7	0.026 (4)	0.037 (4)	0.034 (4)	−0.006 (3)	−0.003 (3)	0.011 (3)
O8	0.033 (3)	0.030 (2)	0.043 (3)	−0.002 (2)	0.011 (2)	0.008 (2)
C9	0.036 (4)	0.034 (4)	0.043 (4)	0.006 (3)	0.010 (4)	0.002 (3)
C10	0.036 (4)	0.046 (5)	0.047 (5)	−0.004 (4)	0.009 (4)	0.010 (4)
C11	0.041 (5)	0.057 (5)	0.048 (5)	0.003 (4)	0.019 (4)	0.019 (4)
C12	0.036 (4)	0.040 (4)	0.044 (4)	−0.002 (4)	0.001 (4)	0.019 (4)
C13	0.035 (4)	0.033 (4)	0.042 (4)	0.000 (3)	0.008 (4)	0.008 (3)
C14	0.031 (4)	0.039 (4)	0.043 (4)	−0.005 (3)	0.004 (3)	0.012 (3)
N15	0.041 (4)	0.022 (3)	0.046 (4)	0.002 (3)	0.001 (3)	−0.003 (3)
N16	0.050 (4)	0.030 (3)	0.057 (4)	−0.001 (3)	0.006 (4)	0.002 (3)
C17	0.048 (5)	0.018 (3)	0.035 (4)	−0.004 (3)	0.003 (4)	0.001 (3)
S18	0.0659 (15)	0.0357 (10)	0.0487 (12)	0.0003 (10)	0.0211 (11)	−0.0008 (9)
C19	0.079 (7)	0.030 (4)	0.067 (6)	−0.003 (4)	0.025 (5)	−0.002 (4)
C20	0.067 (6)	0.038 (5)	0.064 (6)	−0.007 (4)	0.020 (5)	−0.004 (4)
C21	0.088 (8)	0.030 (4)	0.067 (6)	0.000 (5)	0.013 (6)	0.002 (4)
N22	0.042 (4)	0.026 (3)	0.033 (3)	0.001 (3)	0.006 (3)	−0.002 (3)
C23	0.038 (4)	0.036 (4)	0.037 (4)	0.008 (3)	0.002 (3)	−0.004 (3)
C24	0.044 (5)	0.049 (5)	0.028 (4)	0.008 (4)	0.008 (3)	−0.006 (3)
C25	0.032 (4)	0.036 (4)	0.032 (4)	0.011 (3)	0.008 (3)	0.007 (3)
O26	0.034 (3)	0.029 (2)	0.034 (3)	0.002 (2)	0.010 (2)	−0.002 (2)
C27	0.035 (4)	0.038 (4)	0.040 (4)	0.005 (3)	0.012 (3)	0.002 (3)
C28	0.044 (5)	0.040 (4)	0.042 (4)	−0.009 (4)	0.010 (4)	0.010 (4)
O29	0.061 (4)	0.073 (4)	0.048 (3)	−0.019 (3)	0.012 (3)	0.003 (3)
C30	0.052 (5)	0.077 (7)	0.032 (4)	−0.012 (5)	0.013 (4)	−0.013 (4)
C31	0.053 (5)	0.045 (5)	0.036 (4)	−0.002 (4)	0.017 (4)	−0.011 (4)
O1S	0.043 (4)	0.066 (4)	0.067 (4)	−0.005 (3)	−0.004 (3)	0.005 (3)
C2S	0.058 (8)	0.067 (8)	0.25 (2)	−0.008 (7)	0.020 (10)	0.049 (11)
C3S	0.118 (14)	0.139 (15)	0.23 (2)	0.061 (12)	−0.046 (14)	−0.079 (15)

### Geometric parameters (Å, °)

**Table:**

U1—O2	1.772 (5)	C17—S18	1.756 (7)
U1—O3	1.778 (5)	S18—C19	1.812 (9)
U1—O26	2.254 (5)	C19—C20	1.492 (11)
U1—O8	2.285 (5)	C19—H19A	0.9900
U1—O4	2.394 (4)	C19—H19B	0.9900
U1—N22	2.540 (6)	C20—C21	1.306 (12)
U1—N15	2.562 (6)	C20—H20	0.9500
O4—C5	1.417 (10)	C21—H21A	0.9500
O4—H4	0.8600	C21—H21B	0.9500
C5—C6	1.4995 (10)	N22—C23	1.310 (9)
C5—H5A	0.9900	C23—C24	1.406 (11)
C5—H5B	0.9900	C23—H23	0.9500
C6—H6A	0.9800	C24—C31	1.404 (10)
C6—H6B	0.9800	C24—C25	1.429 (10)
C6—H6C	0.9800	C25—O26	1.323 (8)
C7—O8	1.351 (9)	C25—C27	1.376 (10)
C7—C9	1.386 (10)	C27—C28	1.403 (10)
C7—C13	1.419 (9)	C27—H27	0.9500
C9—C10	1.367 (10)	C28—O29	1.314 (9)
C9—H9	0.9500	C28—C30	1.411 (11)
C10—C11	1.406 (10)	O29—H29	0.8600
C10—H10	0.9500	C30—C31	1.337 (11)
C11—C12	1.356 (11)	C30—H30	0.9500
C11—H11	0.9500	C31—H31	0.9500
C12—C13	1.429 (10)	O1S—C2S	1.362 (12)
C12—H12	0.9500	O1S—H1S1	0.8601
C13—C14	1.400 (10)	C2S—C3S	1.54 (2)
C14—N15	1.322 (9)	C2S—H2S1	0.9900
C14—H14	0.9500	C2S—H2S2	0.9900
N15—N16	1.378 (8)	C3S—H3S1	0.9800
N16—C17	1.273 (10)	C3S—H3S2	0.9800
C17—N22	1.422 (9)	C3S—H3S3	0.9800
			
O2—U1—O3	177.8 (2)	C14—N15—U1	124.7 (5)
O2—U1—O26	86.3 (2)	N16—N15—U1	122.1 (5)
O3—U1—O26	92.0 (2)	C17—N16—N15	114.5 (6)
O2—U1—O8	95.3 (2)	N16—C17—N22	123.6 (7)
O3—U1—O8	85.8 (2)	N16—C17—S18	118.2 (5)
O26—U1—O8	160.74 (18)	N22—C17—S18	118.1 (6)
O2—U1—O4	88.96 (18)	C17—S18—C19	100.9 (4)
O3—U1—O4	89.27 (19)	C20—C19—S18	107.9 (6)
O26—U1—O4	79.50 (16)	C20—C19—H19A	110.1
O8—U1—O4	81.34 (16)	S18—C19—H19A	110.1
O2—U1—N22	97.1 (2)	C20—C19—H19B	110.1
O3—U1—N22	83.8 (2)	S18—C19—H19B	110.1
O26—U1—N22	70.73 (18)	H19A—C19—H19B	108.4
O8—U1—N22	127.82 (19)	C21—C20—C19	123.0 (9)
O4—U1—N22	149.10 (18)	C21—C20—H20	118.5
O2—U1—N15	80.9 (2)	C19—C20—H20	118.5
O3—U1—N15	101.3 (2)	C20—C21—H21A	120.0
O26—U1—N15	128.91 (19)	C20—C21—H21B	120.0
O8—U1—N15	70.15 (18)	H21A—C21—H21B	120.0
O4—U1—N15	148.54 (18)	C23—N22—C17	118.7 (6)
N22—U1—N15	62.2 (2)	C23—N22—U1	123.0 (5)
C5—O4—U1	129.2 (5)	C17—N22—U1	117.0 (5)
C5—O4—H4	109.7	N22—C23—C24	127.0 (7)
U1—O4—H4	120.9	N22—C23—H23	116.5
O4—C5—C6	113.3 (8)	C24—C23—H23	116.5
O4—C5—H5A	108.9	C31—C24—C23	118.7 (7)
C6—C5—H5A	108.9	C31—C24—C25	117.0 (7)
O4—C5—H5B	108.9	C23—C24—C25	124.2 (7)
C6—C5—H5B	108.9	O26—C25—C27	119.4 (7)
H5A—C5—H5B	107.7	O26—C25—C24	120.0 (7)
C5—C6—H6A	109.5	C27—C25—C24	120.5 (7)
C5—C6—H6B	109.5	C25—O26—U1	127.8 (4)
H6A—C6—H6B	109.5	C25—C27—C28	119.9 (7)
C5—C6—H6C	109.5	C25—C27—H27	120.0
H6A—C6—H6C	109.5	C28—C27—H27	120.0
H6B—C6—H6C	109.5	O29—C28—C27	122.7 (7)
O8—C7—C9	120.6 (6)	O29—C28—C30	117.5 (7)
O8—C7—C13	118.9 (7)	C27—C28—C30	119.8 (7)
C9—C7—C13	120.5 (7)	C28—O29—H29	112.8
C7—O8—U1	134.8 (4)	C31—C30—C28	119.4 (7)
C10—C9—C7	120.3 (7)	C31—C30—H30	120.3
C10—C9—H9	119.8	C28—C30—H30	120.3
C7—C9—H9	119.8	C30—C31—C24	123.2 (7)
C9—C10—C11	121.2 (8)	C30—C31—H31	118.4
C9—C10—H10	119.4	C24—C31—H31	118.4
C11—C10—H10	119.4	C2S—O1S—H1S1	104.6
C12—C11—C10	119.0 (7)	O1S—C2S—C3S	110.5 (14)
C12—C11—H11	120.5	O1S—C2S—H2S1	109.6
C10—C11—H11	120.5	C3S—C2S—H2S1	109.6
C11—C12—C13	122.1 (7)	O1S—C2S—H2S2	109.6
C11—C12—H12	119.0	C3S—C2S—H2S2	109.6
C13—C12—H12	119.0	H2S1—C2S—H2S2	108.1
C14—C13—C7	124.7 (7)	C2S—C3S—H3S1	109.5
C14—C13—C12	118.2 (7)	C2S—C3S—H3S2	109.5
C7—C13—C12	116.9 (7)	H3S1—C3S—H3S2	109.5
N15—C14—C13	128.6 (7)	C2S—C3S—H3S3	109.5
N15—C14—H14	115.7	H3S1—C3S—H3S3	109.5
C13—C14—H14	115.7	H3S2—C3S—H3S3	109.5
C14—N15—N16	111.7 (6)		
			
O2—U1—O4—C5	−14.1 (7)	N16—C17—S18—C19	−0.2 (7)
O3—U1—O4—C5	164.5 (7)	N22—C17—S18—C19	−176.1 (6)
O26—U1—O4—C5	72.3 (7)	C17—S18—C19—C20	−170.4 (7)
O8—U1—O4—C5	−109.6 (7)	S18—C19—C20—C21	−100.9 (10)
N22—U1—O4—C5	87.9 (8)	N16—C17—N22—C23	167.0 (7)
N15—U1—O4—C5	−84.7 (8)	S18—C17—N22—C23	−17.3 (9)
U1—O4—C5—C6	−134.3 (8)	N16—C17—N22—U1	−0.5 (9)
C9—C7—O8—U1	−138.9 (6)	S18—C17—N22—U1	175.2 (3)
C13—C7—O8—U1	42.9 (10)	O2—U1—N22—C23	120.5 (6)
O2—U1—O8—C7	29.9 (6)	O3—U1—N22—C23	−57.4 (6)
O3—U1—O8—C7	−152.1 (6)	O26—U1—N22—C23	36.9 (5)
O26—U1—O8—C7	123.9 (7)	O8—U1—N22—C23	−137.2 (5)
O4—U1—O8—C7	118.0 (6)	O4—U1—N22—C23	20.7 (8)
N22—U1—O8—C7	−73.3 (7)	N15—U1—N22—C23	−163.7 (6)
N15—U1—O8—C7	−48.5 (6)	O2—U1—N22—C17	−72.5 (5)
O8—C7—C9—C10	−177.8 (7)	O3—U1—N22—C17	109.6 (5)
C13—C7—C9—C10	0.4 (11)	O26—U1—N22—C17	−156.1 (5)
C7—C9—C10—C11	−0.6 (12)	O8—U1—N22—C17	29.8 (6)
C9—C10—C11—C12	1.4 (12)	O4—U1—N22—C17	−172.4 (4)
C10—C11—C12—C13	−2.0 (12)	N15—U1—N22—C17	3.3 (5)
O8—C7—C13—C14	2.1 (11)	C17—N22—C23—C24	177.0 (7)
C9—C7—C13—C14	−176.2 (7)	U1—N22—C23—C24	−16.3 (10)
O8—C7—C13—C12	177.2 (6)	N22—C23—C24—C31	170.9 (7)
C9—C7—C13—C12	−1.0 (11)	N22—C23—C24—C25	−12.0 (12)
C11—C12—C13—C14	177.3 (7)	C31—C24—C25—O26	174.5 (7)
C11—C12—C13—C7	1.8 (11)	C23—C24—C25—O26	−2.7 (11)
C7—C13—C14—N15	−12.0 (13)	C31—C24—C25—C27	−1.7 (11)
C12—C13—C14—N15	172.9 (7)	C23—C24—C25—C27	−178.9 (7)
C13—C14—N15—N16	179.5 (7)	C27—C25—O26—U1	−130.7 (6)
C13—C14—N15—U1	−14.2 (11)	C24—C25—O26—U1	53.1 (8)
O2—U1—N15—C14	−68.2 (6)	O2—U1—O26—C25	−155.6 (6)
O3—U1—N15—C14	112.1 (6)	O3—U1—O26—C25	25.9 (6)
O26—U1—N15—C14	−146.0 (5)	O8—U1—O26—C25	108.9 (7)
O8—U1—N15—C14	30.8 (5)	O4—U1—O26—C25	114.8 (5)
O4—U1—N15—C14	4.5 (8)	N22—U1—O26—C25	−56.8 (5)
N22—U1—N15—C14	−171.2 (6)	N15—U1—O26—C25	−80.4 (6)
O2—U1—N15—N16	96.7 (5)	O26—C25—C27—C28	−176.5 (7)
O3—U1—N15—N16	−83.0 (5)	C24—C25—C27—C28	−0.3 (11)
O26—U1—N15—N16	19.0 (6)	C25—C27—C28—O29	−176.7 (7)
O8—U1—N15—N16	−164.3 (6)	C25—C27—C28—C30	1.5 (12)
O4—U1—N15—N16	169.5 (4)	O29—C28—C30—C31	177.9 (8)
N22—U1—N15—N16	−6.3 (5)	C27—C28—C30—C31	−0.4 (13)
C14—N15—N16—C17	175.2 (7)	C28—C30—C31—C24	−1.8 (14)
U1—N15—N16—C17	8.5 (8)	C23—C24—C31—C30	−179.8 (8)
N15—N16—C17—N22	−5.1 (10)	C25—C24—C31—C30	2.9 (12)
N15—N16—C17—S18	179.3 (5)		

### Hydrogen-bond geometry (Å, °)

**Table:**

*D*—H···*A*	*D*—H	H···*A*	*D*···*A*	*D*—H···*A*
O4—H4···O8^i^	0.86	1.75	2.601 (7)	173.
O29—H29···O1S	0.86	1.79	2.646 (9)	171.
O1S—H1S1···O26^ii^	0.86	1.87	2.718 (8)	169.
C23—H23···O2^iii^	0.95	2.49	3.395 (9)	158.
C27—H27···O1S	0.95	2.51	3.189 (10)	128.

Symmetry codes: (i) −*x*+2, −*y*, −*z*; (ii) −*x*+1, −*y*, −*z*; (iii) *x*, −*y*+1/2, *z*+1/2.
